# Using Machine Learning to Predict Invasive Bacterial Infections in Young Febrile Infants Visiting the Emergency Department

**DOI:** 10.3390/jcm10091875

**Published:** 2021-04-26

**Authors:** I-Min Chiu, Chi-Yung Cheng, Wun-Huei Zeng, Ying-Hsien Huang, Chun-Hung Richard Lin

**Affiliations:** 1 Department of Emergency Medicine, Kaohsiung Chang Gung Memorial Hospital, Kaohsiung 833, Taiwan; outofray@hotmail.com (I.-M.C.); qzsecawsxd@cgmh.org.tw (C.-Y.C.); 2 Department of Computer Science and Engineering, National Sun Yat-Sen University, Kaohsiung 804, Taiwan; ninefour@g-mail.nsysu.edu.tw; 3 Department of Pediatrics, Kaohsiung Chang Gung Memorial Hospital, Kaohsiung 833, Taiwan; yhhuang@cgmh.org.tw

**Keywords:** machine learning, invasive bacterial infection, young infant fever, emergency department

## Abstract

Background: The aim of this study was to develop and evaluate a machine learning (ML) model to predict invasive bacterial infections (IBIs) in young febrile infants visiting the emergency department (ED). Methods: This retrospective study was conducted in the EDs of three medical centers across Taiwan from 2011 to 2018. We included patients age in 0–60 days who were visiting the ED with clinical symptoms of fever. We developed three different ML algorithms, including logistic regression (LR), supportive vector machine (SVM), and extreme gradient boosting (XGboost), comparing their performance at predicting IBIs to a previous validated score system (IBI score). Results: During the study period, 4211 patients were included, where 126 (3.1%) had IBI. A total of eight, five, and seven features were used in the LR, SVM, and XGboost through the feature selection process, respectively. The ML models can achieve a better AUROC value when predicting IBIs in young infants compared with the IBI score (LR: 0.85 vs. SVM: 0.84 vs. XGBoost: 0.85 vs. IBI score: 0.70, *p*-value < 0.001). Using a cost sensitive learning algorithm, all ML models showed better specificity in predicting IBIs at a 90% sensitivity level compared to an IBI score > 2 (LR: 0.59 vs. SVM: 0.60 vs. XGBoost: 0.57 vs. IBI score >2: 0.43, *p*-value < 0.001). Conclusions: All ML models developed in this study outperformed the traditional scoring system in stratifying low-risk febrile infants after the standardized sensitivity level.

## 1. Introduction

Febrile infants ≤ 60 days of age are prone to contracting serious bacterial infections (SBIs). However, no reliable physical examination findings or specialized routine laboratory investigations exist that can aid a clinician in differentiating an SBI from benign viral infections. Although several criteria (Boston, Philadelphia, and Rochester) proposed to stratify low-risk patients [1,2,3] are regarded as commonly used assessment tools, the development of novel diagnostic tools and changes in epidemiology [4] have led to a decrease in the adherence to these guidelines by clinical physicians [5,6,7]. This issue urged the formulation of a new management guideline to effectuate improvements on the several protocols proposed earlier [8,9].

Urinary tract infections (UTIs) constitute the majority of SBIs in febrile infants [10,11,12]. Urinalysis is a highly noninvasive and sensitive method that facilitates diagnosis for UTI and is performed in an emergency department (ED) [13]. Therefore, focusing on the specific type of infection was more important to ED physicians than the generalized concept of SBIs, in terms of evaluation and management. Consequently, the term “invasive bacterial infection (IBI)”, including bacteremia and bacterial meningitis, which were more likely to result in adverse outcomes [14], gained more popularity [15,16,17].

Several studies focusing on developing prediction models for identifying young infants at low-risk for IBIs have been reported over the past few years [18,19]. In 2014, Mintegi et al. adopted various parameters, including clinical appearance, age, urinalysis results, and laboratory examinations such as white blood cell (WBC) count, c-reactive protein (CRP), and procalcitonin tests, to achieve better prediction performance than that of existing protocols [19]. As procalcitonin and CRP levels are not routinely obtained in general practice, Aronson et al. further proposed a prediction model called “IBI score” that employed only four characteristics (age, temperature, urinalysis results, and absolute neutrophil count) to enhance the generalizability of the model [20].

Machine learning (ML), as an application of artificial intelligence using computer-based algorithms that can directly learn and identify trends from a dataset, has exhibited promising results on the diagnosis of sepsis and predicted clinical outcomes in adult patients in the ED [21,22,23,24]. However, to the best of our knowledge, a study on the prediction of IBIs in young infants based on ML algorithms has not been conducted. Thus, the objective of this study was to develop and validate an ML model to predict the possibility of IBIs among young febrile infants based on certain clinical parameters.

## 2. Materials and Methods

### 2.1. Study Design

This paper details a retrospective study conducted in the EDs of three medical centers across Taiwan, from 1 January 2011 to 31 December 2018. All three hospitals are branches of the same healthcare system and are geographically well dispersed. The three hospitals were located in northern, middle east, and southern Taiwan with annual pediatric ED visit of 30,000, 10,000, and 20,000, respectively. The study was approved by the institutional review board of the Chang Gung Medical Foundation (IRB number: 202001949B0, Date of Approval: 24 November 2020). The patient and physician records and information were anonymized and deidentified prior to the analysis.

### 2.2. Patient Population and Data Collection

All patients aged 0–60 days visiting the ED with clinical symptoms of fever in the period mentioned above were included as subjects in this study. Patients with subjectively described fever or elevated body temperature noted at ED admission were both included. Patients with missing laboratory data, uncertain test results (due to the inability to obtain blood or cerebral spinal fluid (CSF) culture reports), or complex chronic conditions were excluded from the study population, along with transfers from other hospitals and premature infants [25]. The parameters collected were age, sex, vital signs during the ED visit, and laboratory test results. In this study, the growth of a pathogen observed in the blood and CSF cultures was considered to be an IBI [16].

### 2.3. Feature Selection

Previous statistical analyses focused on the association between features and outcomes, in which *p*-values were used as a measure of association [26,27]. In contrast, we determined the order of importance among the features, using a forward stepwise method to control overfitting, and included the most suitable subsets of these features. This method used a sequence of steps so that only one feature can enter at a time. In most cases, the process converged to a subset of features that helped the ML model yield the best prediction performance, which typically used the value of area under the receiver-operating characteristic curve [AUROC] [28] as an evaluation tool in an imbalanced dataset. The best feature subset of the AUROC value was used for ML training.

### 2.4. Machine Learning Models

We developed and trained three ML algorithms in this study, i.e., logistic regression (LR), support vector machine (SVM), and extreme gradient boosting (XGBoost), using the TensorFlow 2.3 package. LR is a predictive analysis algorithm based on the concept of probability, which uses the sigmoid function to derive the predicted output for classification. SVM is another ML model commonly used for solving classification problems. The SVM constructs a set of hyperplanes in a higher-dimensional space to obtain the largest distance to the nearest training data point of any class. The larger the distance of this margin achieved, the lower the generalization error [29]. This method usually performed better than LR. In contrast, XGBoost is an ensemble learning algorithm proposed for sparse data and weighted quantile sketch. XGBoost was developed to solve real-world imbalanced data problems using the stacking of decision trees [30].

As the probability of IBI was low in the general population visiting the ED, IBI prediction was considered as an imbalanced class problem. In this study, we replaced the loss function of the cross entropy, typically used in ML classification problem, with the cost sensitive matrix. The essence of the cost sensitive method helps the ML model make the optimal decision while accounting for the cost of the prediction error during training to avoid deviations in the prediction performance to the non-IBI group [31].

### 2.5. Model Evaluation and Statistical Analysis

All included patients were randomly assigned into five groups which contained 20% of patients, and the developed ML models were assessed using 5-fold cross validation, which means one group was regarded as a validation set and the other four were regarded as training sets each time. The result of model performance was then compared to the IBI score devised by Aronson et al. [20] to predict IBIs in young febrile infants ≤ 60 days old. The IBI score were developed statistically and ranged from 0–10, the higher scores indicated higher risk of IBIs (Table 1). Other statistically derived scoring systems in previous studies, such as the step-by-step method [19] and lab-score [18], were not included for comparison, as the procalcitonin test was not conducted for most of the patients in this study.

We estimated the AUROC curve with sensitivity and specificity as the performance parameters. For each model, we adjusted the class weight on the outcome prediction to prioritize sensitivity at a level of 90% and compared the predicted results with the IBI score at the same level (IBI score ≥ 2). The above statistical analyses were performed using Python 3.8 with the Scikit-learn 0.22.2 package [32].

## 3. Results

In the study period, 4211 patients were included for analysis. Patients’ distribution and demographics in three hospitals were shown in Table 2. A total of 126 (3.1%) patients had IBI; among them, 117 (2.8%) and 18 (0.4%) patients had bacteremia and bacterial meningitis, respectively. The clinical characteristics of the patients with and without IBI are demonstrated in Table 3. The factors significantly associated with IBI were age (31 (20–43) vs. 36 (23–50) days, *p* = 0.001), temperature at triage (38.4 (37.9–38.9) vs. 37.7 (37.1–38.3) °C, *p* < 0.001), heart rate (177 (161–189) vs. 159 (143–175) per minute, *p* < 0.001), and laboratory tests, namely hemoglobin, platelet, C-reactive protein, differential count of WBC, including neutrophil, band, eosinophil, lymphocyte, and abnormal urine tests. The IBI score was significantly higher in patients with IBI than that of those without IBI (4 (2–6) vs. 2 (0–4), *p* < 0.001).

The results of the forward stepwise feature selection method performed for each of the ML models are described in Table 4. Among the three models, SVM used the least number of features to achieve the highest AUROC value (0.84 ± 0.03). Among these, the common shared features were CRP and band. The neutrophil count and heart rate were also adopted in two out of three models.

**Figure 1:**
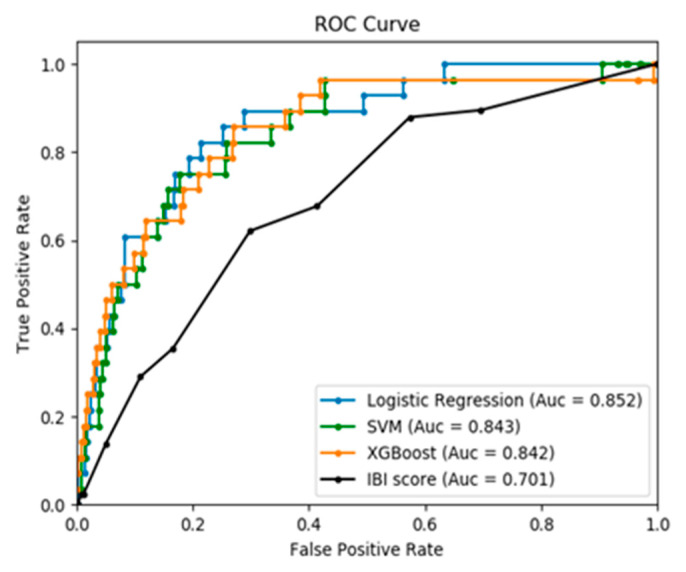
Receiver-operating characteristic (ROC) curves of the IBI score and the developed machine learning models on the validation set; SVM = Support vector machine; XGBoost = Extreme gradient boosting.

Analysis of variance (ANOVA) was performed to compare the performance of the ML models with the IBI score (Table 5). All the ML models outperformed the IBI score in the prediction of IBI in young febrile infants based on the AUROC level. The receiver operating characteristic curves of all the evaluated models are illustrated in Figure 1. There was no significant difference in the AUROC value among the selected ML models in the post-hoc test. After class-weight adjustment, the ML models demonstrated no statistical difference in sensitivity of IBI prediction compared with using an IBI score of ≥2 as a predictor. In contrast, the ML models displayed better specificity levels than those of the IBI score. Among the three models, the specificity for IBI prediction was slightly better in LR and SVM than XGBoost, with significant differences in post-hoc analysis.

## 4. Discussion

Ramgopal et al. [33] developed various ML models, including the LR, SVM, RF, and single-layer neural networks, to predict SBIs in young febrile infants. With a 9.3% incidence of SBIs in the included patients, the ML techniques were able to produce better results than those of previous scoring systems. In this study, we extended the above-mentioned study to predict IBIs in young febrile infants visiting the ED, as bacteremia and bacterial meningitis are difficult to diagnose in EDs, and UTIs can usually be diagnosed with a urinalysis. This finding showed that urinalysis is the most important feature for prediction of SBI in that study, while UTIs account for 88.4% in all SBI patients. Our study focused on predicting IBIs and showed that ML was still able to achieve better AUROC values when predicting IBIs compared to the IBI score.

Another difference between our study and Ramgopal et al. is that we did not include procalcitonin as a feature in developing ML models. We also did not exclude patients of critical appearance. The major reason for the design is we tend to keep as many patients as possible to avoid model bias on clinical application. The difference can be noticed as only 20.0% patients encountered were included in Ramgopal’s study, and most of the patients encountered were included in our study.

In this study, we used a cost sensitive technique to manage the imbalanced data problem. Cost-sensitive algorithms are a subfield of ML and are commonly adopted during the training of ML models as imbalanced data is frequently observed in real-world scenarios. In basic supervised ML models, the algorithm tends to lean toward the majority of the population, which affects the application of the model. For example, if disease X occurred in only 1% of the study population, the model can easily predict that no one in the population has disease X as this would be true in 99% of the cases. Cost-sensitive methods were hence developed to solve this problem by assigning more cost when the model predicted trends in the minority population inaccurately during training [34,35].

With aid from practical ML applications, the result of this study can reduce unnecessary admissions and prolonged antibiotic use in low-risk patients. In clinical practices, physicians can prioritize high sensitivity to differentiate IBIs from benign viral infections in young febrile infants. With the low prevalence of IBIs among young infants, previous models can only achieve a 46.9–52.0% specificity in the prediction of IBIs at a 90% sensitivity level [9,20]. Using the cost sensitive algorithm, we were able to set the sensitivity of the prediction models to 90% and evaluate their performance on the specificity at a similar standard. The results showed that all three models outperformed the IBI score at stratifying low-risk patients (Table 5).

A total of eight, five, and seven features were used in the LR, SVM, and XGboost, respectively, to achieve optimal AUROC levels during training through the stepwise feature selection method (Table 4). The common features extracted in the ML models based on the adopted parameters were of reasonable relevance, as CRP, neutrophil, and band counts were reported to have a positive correlation with bacterial infections in young infants [17,36]. Compared to the IBI score, which consisted of four different parameters to predict IBI, commonly shared parameters in ML models were ANC and neutrophil. Age and temperature were only adopted in LR. SVM and XGBoost did not acquire these two features and were still able to achieve similar AUC values as LR. Increase of temperature was one of the vital signs that correlated with pediatric sepsis. However, a recent study showed that although IBI was more likely with higher temperatures, degree of fever should not be used in the risk stratification of febrile infants [37]. On the other hand, bacteremia was associated with UTIs in 4–10% of febrile infants [38]. It was reasonable to include abnormal urinalysis in ML models to predict IBIs in febrile infants. One possible cause that all of the ML models did not use abnormal urinalysis as a ML feature is that we adopted CRP as one of the features, which was proved to be able to stratify risk in febrile infants with leukocyturia in multiple studies [39,40].

A multicenter study conducted in 2017 discovered that none of the parameters in a complete blood cell count were able to efficiently predict IBIs in young infants [16]. In this study, we used the stepwise feature-selection method not only to demonstrate the real-world applicability of the extracted features, but also to attempt to obtain the highest possible accuracy for each of the N-feature choices among all of the feature sets. The idea behind deploying a forward stepwise regression for feature selection, instead of a regression analysis, was to achieve better performance from the ML models using feature combinations, rather than combining statistically significant features. Consequently, we were also able to enumerate the selected features in an orderly manner based on their level of importance to the model.

Although ML models can directly learn from the dataset and improve the diagnosis and outcome prediction in several areas across medicine, the black-box nature of each network can present challenges when applying or extending the proposed models to clinical practice. In this study, we attempted to overcome this problem using the stepwise feature-selection method and selecting features with better generalizability, such as age, sex, vital signs, and laboratory test results. We speculate that the results of our study can provide a reference for the application of ML models across various healthcare systems. However, we observed a limitation. As this is a retrospective study, data could only be collected based on past medical records. More recent laboratory tests that may have direct links to pediatric sepsis, such as procalcitonin or lactate [41,42], could not be included as a large amount of data was not obtained during the ED visit. This situation limited the comparison of the developed ML models with other recently reported prediction models.

## 5. Conclusions

In this study, we developed and evaluated the performance of three ML models, including LR, SVM, and XGBoost, to predict IBIs in young febrile infants in the ED. All the ML models outperformed traditional scoring systems in stratifying low-risk febrile infants after standardized sensitivity level. Among the three, the SVM algorithm exploited the least number of features to achieve the optimal result.

## Figures and Tables

**Table 1:** Invasive bacterial infection (IBI) score.

Predictor	Points ^a^
Age < 21 days old	1
Highest temperature in the ED 38.0–38.4 °C	2
Highest temperature in the ED ≥ 38.4 °C	4
Abnormal urinalysis result ^b^	3
ANC ≥ 5185 cells per µL	2

^a^ Total possible scores ranged from 0 to 10. ^b^ Urine dipstick with positive leukocyte esterase or positive nitrites or urine microscopy with >5 WBCs per high-power field or >5 WBCs per mm^3^ on enhanced urinalysis.

**Table 2:** Distribution and demographics of patients in three examined hospitals.

	Northern Hospital Mean (SD)/*n*(%)	Middle West Hospital Mean (SD)/*n*(%)	Southern Hospital Mean (SD)/*n*(%)	*p*-Value
Total number of patients	2653	168	1390	
Age, days-old	32 (18.0)	31 (17.5)	32 (18.1)	0.504
Male	1552 (58.5)	91 (54.2)	819 (58.9)	0.497
IBI	82 (3.1)	3 (1.8)	41 (2.9)	0.625
Bacteremia	76 (2.9)	2 (1.2)	39 (2.8)	0.439
Bacterial Meningitis	14 (0.5)	1 (0.6)	3 (0.2)	0.333

**Table 3:** Demographic and clinical characteristic comparison between patients with and without invasive bacterial infections.

	With IBI (*n* = 126)	Without IBI (*n* = 4085)	*p*-Value
Age, d, median (IQR)	31 (20–43)	36 (23–50)	0.001
Male sex, *n* (%)	78 (61.9)	2384 (58.4)	0.463
Vital signs			
Triage temperature, median (IQR)	38.4 (37.9–38.9)	37.7 (37.1–38.3)	<0.001
Highest ED temperature, median (IQR)	38.6 (38.0–39.1)	37.8 (37.2–38.4)	<0.001
Triage HR, median (IQR)	177 (161–189)	159 (143–175)	<0.001
Laboratory test			
WBC, median (IQR)	10.9 (6.4–14.1)	11.2 (8.2–13.4)	0.462
Hb, median (IQR)	11.6 (9.8–12.8)	12.3 (10.2–14.2)	<0.001
Platelet, median (IQR)	365 (285–441)	389 (302–458)	0.027
Neutrophil, median (IQR)	55.7 (43.7–69.1)	37.1 (24–49)	<0.001
Band, mean	0 (0–1.5)	0 (0)	<0.001
Eosinophil, median (IQR)	0.8 (0–2.0)	2 (1–4)	<0.001
Lymphocyte, median (IQR)	33.9 (22.8–45.1)	47.5 (35.2–60.0)	<0.001
ANC, median (IQR)	6397 (2828–8795)	4368 (2112–5546)	<0.001
CRP, median (IQR)	35.2 (2.4–47.6)	0.8 (0–5.7)	<0.001
Abnormal urine test, *n* (%)	61 (48.6)	740 (18.2)	<0.001
IBI score, median (IQR)	4 (2–6)	2 (0–4)	<0.001
IBI ≥ 2, *n* (%)	110 (87.3)	2345 (57.4)	<0.001

**Table 4:** Adopted features in the developed machine learning (ML) models using the stepwise feature selection method.

	Logistic Regression	SVM	XGBoost
1st	Neutrophil	CRP	Eosinophil
2nd	CRP	Heart Rate	Band
3rd	Lymphocyte	Neutrophil	WBC
4th	Basophil	Basophil	CRP
5th	Band	Band	Heart Rate
6th	Platelet		ANC
7th	Age		Monocyte
8th	Temperature		

**Table 5:** ANOVA analysis of outcome prediction using the IBI score and developed ML models.

Outcome, Mean (SD)	IBI Score	LR	SVM	XGBoost	*p*-Value
AUROC	0.70 (0.03) *	0.85 (0.04)	0.84 (0.03)	0.84 (0.03)	<0.001
IBI score ≥ 2					
Sensitivity	0.85 (0.06)	0.90 (0.07)	0.91 (0.07)	0.90 (0.08)	0.219
Specificity	0.43(0.01) *	0.59 (0.02) **	0.60 (0.03) **	0.57 (0.02)	<0.001

* significantly lower than the outcomes of other models in the post-hoc test. ** significantly higher than the outcomes of other models in the post-hoc test.

## Data Availability

Data was obtained from Chang Gung Medical Foundation and are available from corresponding author with the permission of Chang Gung Medical Foundation.
